# Dermoscopy as a Tool for Identifying Potentially Metastatic Thin Melanoma: A Clinical–Dermoscopic and Histopathological Case–Control Study

**DOI:** 10.3390/cancers16071394

**Published:** 2024-04-01

**Authors:** Vincenzo De Giorgi, Flavia Silvestri, Giovanni Cecchi, Federico Venturi, Biancamaria Zuccaro, Gabriella Perillo, Federica Cosso, Vincenza Maio, Sara Simi, Pietro Antonini, Serena Pillozzi, Lorenzo Antonuzzo, Daniela Massi, Laura Doni

**Affiliations:** 1 Section of Dermatology, Department of Health Sciences, University of Florence, 50121 Florence, Italy; flavia.silvestri25@gmail.com (F.S.); giovanni.cecchi1@unifi.it (G.C.); federico.venturi@unifi.it (F.V.); biancamaria.zuccaro@unifi.it (B.Z.); gabriella.perillo@unifi.it (G.P.); 2 Cancer Research “Attilia Pofferi” Foundation, 51100 Pistoia, Italy; 3 Medical Oncology Unit, Careggi University Hospital, 50134 Florence, Italy; federica.cosso@unifi.it (F.C.); serena.pillozzi@unifi.it (S.P.); lorenzo.antonuzzo@unifi.it (L.A.); doni.laura@mail.com (L.D.); 4 Department of Experimental and Clinical Medicine, University of Florence, 50121 Florence, Italy; 5 Section of Pathology, Department of Health Sciences, University of Florence, 50121 Florence, Italy; vincenza.maio@unifi.it (V.M.); sara.simi@unifi.it (S.S.); pietro.antonini@studenti.univr.it (P.A.); daniela.massi@unifi.it (D.M.); 6 Section of Pathology, Department of Diagnostic and Public Health, University of Verona, 37129 Verona, Italy

**Keywords:** dermatoscopy, dermoscopy, skin cancer, early-stage melanoma, tumor thickness, metastasis

## Abstract

**Simple Summary:**

Due to their high incidence, thin melanomas represent a significant portion of all melanoma-related deaths (30%). In this retrospective case–control study, metastasizing thin melanomas (≤0.8 mm) showed at least one atypical clinical–dermoscopic feature (diameter > 10 mm, at least three colors, regression structures, atypical vascular pattern and absence of a pigment network) and at least one conventional adverse histopathological feature (regression, dermal mitoses, vertical growth phase and ulceration). In thin melanomas, the identification of specific clinical–dermoscopic features (statistically significant) coupled with adverse histopathological features may suggest an early detection of potentially metastatic patients who require prolonged and close monitoring.

**Abstract:**

Despite being early-stage tumors, thin cutaneous melanomas contribute significantly to mortality and have a rising incidence. A retrospective case–control study was performed to identify clinical–dermoscopic and histopathological variables linked to local and distant metastases in melanomas ≤0.8 mm. Data from 1 January 2000 to 22 June 2022 were analyzed from two Italian skin cancer referral centers. Sixteen patients with ≤0.8 mm melanomas developing metastases were studied compared to controls without metastases over 5 years. Statistical analysis involved Pearson’s chi-squared test or Fisher’s exact test. Of the 1396 cases, 1.1% progressed. The median diagnosis age was 49 (range 28–83), with 56.3% men and 43.7% women. The torso was the primary tumor site (43.7%). Clinically, lesions were pigmented (>10 mm diameter: 73.3%, ≥3 colors: 80%). Dermoscopically, the common features were white patches (73.3%), atypical vascular patterns (66.5%), blue-gray areas (60%) and absent pigment networks (60%). Histopathologically, all cases had adverse features like regression (87.4%), dermal mitoses (50%), a vertical growth phase (62.5%) and ulceration (12.5%). These findings were statistically significant compared to controls (*p* < 0.05). In ≤0.8 mm melanomas, specific clinical–dermoscopic traits might indicate higher metastatic potential when paired with adverse histopathological features.

## 1. Introduction

The incidence of melanoma is rapidly increasing worldwide, and it is one of the most frequent tumors affecting young people. In Italy, it represents the second most frequent tumor in men < 50 years and the third most frequent tumor in women < 50 years, with an estimated lifetime risk of developing melanoma of 1.5% for men and 1.2% for women. In contrast, the melanoma mortality rate has been stable worldwide.

Several studies have shown that the increased incidence of new invasive lesions is due largely to thin melanomas (Breslow thickness ≤ 1 mm), which represent 70–80% of newly diagnosed lesions. Dermoscopy and more recent non-invasive diagnostic techniques such as reflectance confocal microscopy, which have become increasingly predominant in dermatologists’ daily practice, have a role in the overdiagnosis and earlier detection of thin melanomas in recent years. An increased consciousness among people of skin cancers, along with wide-reaching melanoma screening campaigns, encourages early facilitated access to healthcare services.

Nonetheless, the factors playing a specific role in the rising incidence of thin lesions are not yet completely understood. Furthermore, melanoma mortality rates have generally remained stable [1,2,3,4].

According to the current staging system of the American Joint Committee on Cancer (AJCC) (8th Edition), melanoma-specific survival rates for stage IA are 99% at 5 years and 98% at 10 years [5], suggesting a favorable prognosis for patients with pT1 tumors. Only a small proportion (5–20%) of these melanomas results in regional or distant metastases, sometimes causing death (5%). However, thin melanomas represent a significant portion of all melanoma-related deaths (30%) due to their high incidence [4,6,7,8].

The aim of this retrospective case–control study was to provide our 22 years of experience with metastatic thin melanomas (MTMs) and to report their demographic, clinical, dermoscopic and histopathological characteristics.

Most of these patients have been diagnosed with a low-risk melanoma; however, their clinical outcome was comparable to that of thicker melanomas, although they are considered highly curable tumors. Such observations highlight the variable risk associated with TM and the challenging definition and management of this subset of patients, first of all in terms of accurate staging. In the current national and international guidelines, there is no clear evidence about the optimal follow-up management of patients diagnosed with TM, in particular in terms of close monitoring in order to ensure early detection of a potential disease progression and in terms of long-term follow-up strategies in order to identify late-onset metastasis development in relapsed high-risk patients in respect to cost-effectiveness in a real-world setting.

The early detection of metastatic disease is particularly crucial for this subgroup of patients as the extraordinary progress in systemic therapies experienced in the last decade has completely changed the current metastatic patient prognosis, resulting in improved clinical outcomes and prolonged survival rates.

For these reasons, we compared the clinical, dermoscopic and histopathological features of a series of MTMs with those of a non-MTM control group in order to identify prognostic features.

In the end, the identification of reliable prognostic factors through the evaluation of easy-to-access tumor characteristics, such as clinical, dermoscopic and pathological features, along with a better comprehension of the mechanisms of progression of cutaneous melanoma, may help physicians obtain more precise and tailored approaches in the management of this subset of patients.

## 2. Methods

An observational and retrospective case–control study was conducted from January 2000 to June 2022 at two referral centers (Dermatology Unit, Azienda USL Toscana Centro and Medical Oncology Unit, Careggi University Hospital, Florence, Italy). These centers comply with a policy of photographing all equivocal lesions prior to biopsy or excision. Cases included consecutive patients histologically diagnosed as primary cutaneous melanoma with a Breslow thickness ≤ 0.8 mm who developed regional and/or distant metastases. Controls were consecutive patients histologically diagnosed as having a primary cutaneous melanoma with a Breslow thickness ≤ 0.8 mm who did not develop regional and/or distant metastases with at least 5 years of follow-up. Participants consented to the use of their de-identified information and images for research purposes. Patients with other malignancies or multiple primary melanomas were excluded. 

Patients’ primary tumor data were retrospectively retrieved from two different databases and included demographic, clinical, dermoscopic, histopathological and conventional parameters. All cases were re-staged according to the current AJCC Staging system (8th Edition). The original histopathological glass slides were available for review in 14 out of 16 cases. Histopathology review was conducted by two dermatopathologists specialized in the diagnosis of skin cancer. 

A handheld dermatoscope (Heine Delta 20; Heine Optotechnick, Herrsching, Germany) was used for dermoscopic examination. Both clinical and dermoscopic images of all lesions were captured with a high-resolution compact digital camera (Olympus Digital model no. E-520, 7.1-megapixel digital photo camera with a 3.8 optical zoom lens, focal length of 28–105 mm in a 35 mm format and a maximum lens aperture of f/2.8–f/5.8). 

In vivo dermoscopic image assessment was carried out via Dermaphot (Heine Optotechnick), which connects a dermatoscope to the camera to generate reproducible, high-quality dermoscopic pictures at 10-fold magnification in the joint photographic expert group (JPEG) format. These clinical and dermoscopic images and the data were stored on a standard Windows-based personal computer. A group of investigators with expertise in dermoscopy from the Dermatology Department (University of Florence, Florence, Italy) analyzed (blinded for follow-up and diagnosis) the archived digital dermoscopic images and completed a printed questionnaire to categorize the lesions according to the typical dermoscopic pattern analysis. The dermatologists had comparable levels of training and experience in dermatology with more than 5 years of practice in dermoscopy. The dermatoscopic criteria applied were those indicated in internationally proposed classifications and algorithms. 

Treatment of the primary tumor consisted of surgical excision followed by wider excision based on the Breslow thickness (histologically confirmed 0.5 cm margins for in situ melanoma and 1 cm margins for pT1 lesions in healthy tissue). All cases were discussed in a multidisciplinary tumor board, which approved a therapeutic plan, and the choice of sentinel lymph node biopsy (SLNB) was discussed with patients when needed (according to the AJCC staging system edition of the time).

The disease progression data (including anatomical site), type of treatment and clinical outcome were analyzed. Melanoma metastases that occurred at the time of diagnosis or during follow-up were classified as regional if they were in lymph nodes and as distant if they were visceral, cutaneous (at distant sites from primary tumor), bone or brain metastases. All local and regional metastases were diagnosed histologically, while distant metastases were instrumentally detected and/or confirmed histopathologically. 

Statistical analysis was performed with IBM SPSS software (version 25, IBM Corp., Armonk, NY, USA). The association between categorical variables was evaluated with Pearson’s chi-squared test or Fisher’s exact test, as appropriate. A two-tailed *p*-value of less than 0.05 constituted statistical significance.

## 3. Results

Between January 2000 and June 2022, 1396/1864 (75%) patients diagnosed with melanoma were histopathologically diagnosed as in situ or thin melanoma with a Breslow thickness ≤ 0.8 mm. Overall, 16/1396 (1.1%) melanoma patients experienced disease progression, including regional nodal or distant metastasis. Four of them (three females and one male) presented metastasis as the first manifestation of the disease. Among them, two patients showed regional nodal metastases, while two additional patients showed distant metastases, including central nervous system (CNS) involvement. In four cases, the Breslow thickness was ≤0.6 mm. A control group of 100 consecutively selected non-metastatic thin melanoma patients with at least 5 years of follow-up was obtained from the same database and in the same time period. Patient and tumor characteristics for both groups are summarized in Table 1. In the case group, the median age at diagnosis was 49 years (range 28–83 years), and 56.3% were aged under 50 years. The genders were equally distributed with nine men (56.3%) and seven women (43.7%). 

The most frequent anatomical site of the primary melanoma was the torso (n = 7, 43.7%), followed by upper limbs (n = 6, 37.5%), lower limbs (n = 2, 12.5%) and the head/neck region (n = 1, 6.3%).

**Figure 1:**
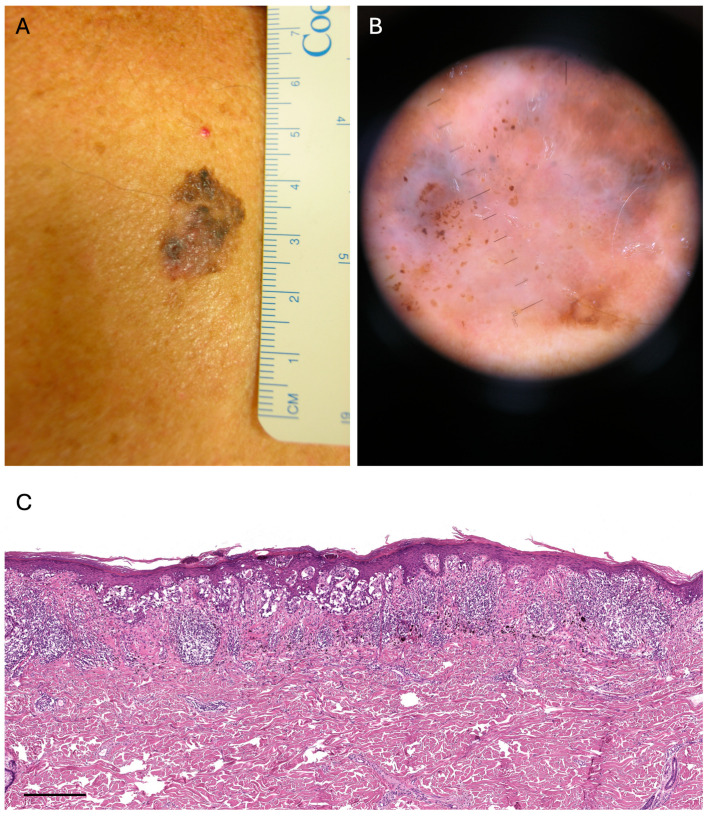
(**A**). Clinical image of a pT1a melanoma: >10 mm diameter (Breslow 0.6 mm, no ulceration) on the torso of a 62-year-old male patient. (**B**). Dermoscopy of the lesion: absence of pigment network, >3 colors, atypical vascular pattern and peripheral blue-white veil. (**C**). Thin superficial spreading melanoma/low-CSD melanoma associated with regression (partial replacement of the tumor with variably vascular fibrous tissue, accompanied by pigment-laden macrophages and chronic inflammation), original magnification 4×, scale bar 250 μm, hematoxylin and eosin stain.

**Figure 2:**
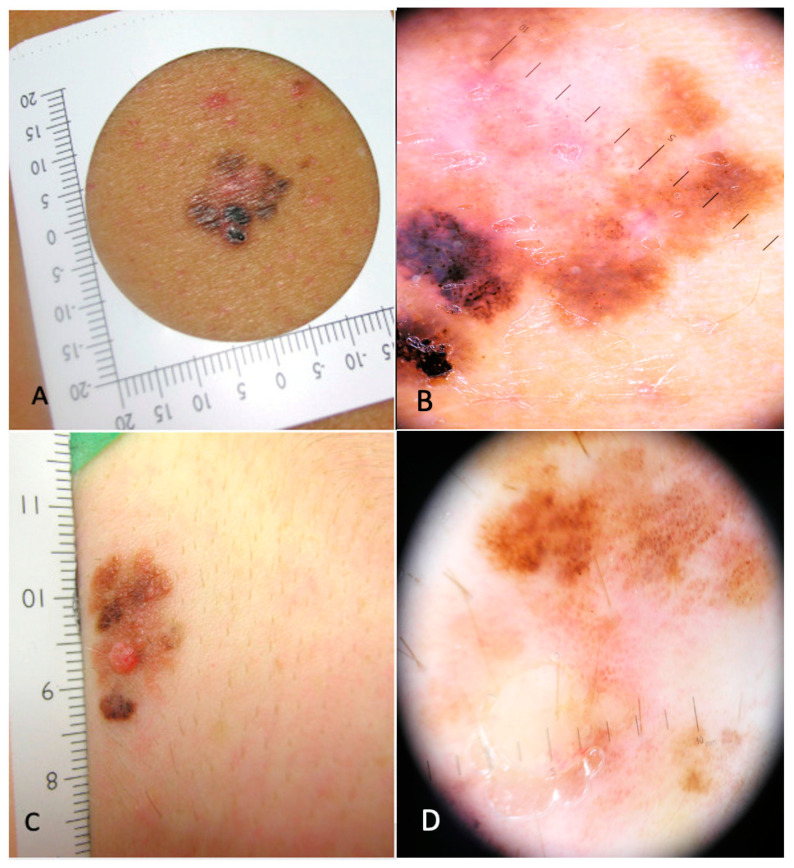
(**A**). Clinical image of a pT1a melanoma: >10 mm diameter (Breslow 0.2 mm, no ulceration) on the torso of a 40-year-old male patient. (**B**). Dermoscopy of the lesion: absence of pigment network, >3 colors, atypical vascular pattern and central white patch. (**C**). Clinical image of a pT1a melanoma: >10 mm diameter (Breslow 0.6 mm, no ulceration) on the neck of a 29-year-old female patient. (**D**). Dermoscopy of the lesion: absence of pigment network, >3 colors, atypical vascular pattern, diffuse white patches.

We retrieved 16 clinical and dermoscopic images from the case group (MTM) (Figure 1 and Figure 2) and 100 clinical and dermoscopic images from the control group (non-MTM). The clinical and dermoscopic characteristics of the MTMs and the non-MTM patients are summarized in Table 2. 

Clinically, all the MTM lesions were pigmented and dyschromic, with a diameter > 10 mm in 11 cases (73.3%, *p* = 0.002) and at least three colors (black, brown, gray, blue, red, white) in 12 cases (80%, *p* ≤ 0.001). On the contrary, in the control group, only 3% of the lesions had more than three colors and only 29% had a size of greater than 10 mm.

Dermoscopic features such as blue-gray areas, white patches and atypical vascular patterns were present in 9 (60%, *p* = 0.038), 11 (73.3%, *p* = 0.055) and 10 (66.5%, *p* ≤ 0.001) lesions, respectively, in the case group (MTM). Meanwhile, in the controls (non-MTM), the same dermoscopic parameters typical of thick lesions appeared with significantly lower percentages: blue-gray areas at 27%, white patches at 43% and atypical vascular patterns at only 15% (Table 2). 

Furthermore, other dermoscopic characteristics such as the blue-white veil and the absence of a pigment network were present in 20% (*p* = 0.079) and 60% (*p* = 0.001) of cases, respectively, while in the control group, the blue-white veil was recorded in 5% and the absence of pigment network in 15% of controls.

Histopathologically, in the case group, there were two regressed melanomas with a residual intraepidermal/in situ component. Among MTM patients, there were 14 (87.5%) invasive SSM/low-CSD melanomas according to the current WHO 4th edition [9], including 5/14 with nevus remnants. The average Breslow thickness was 0.6 mm (range 0.2–0.8 mm), and 56.2% of cases showed a Breslow thickness ≥ 0.5 mm (*p* ≤ 0.001). Ulceration was found in two cases (12.5%, *p* = 0.044). VGP was demonstrated in 10 cases (62.5%, *p* = 0.002). The mitotic rate ranged 1–4 mitoses/mm^2^ in invasive melanoma. Specifically, the number of mitoses/mm^2^ was 4 in 1 case (6.3%, *p* ≤ 0.001), 3 in 2 cases (12.5%), 2 in 1 case (6.3%), 1 in 4 cases (25%) and 0 in 5 cases (31.5% (*p* ≤ 0.001) (Table 1). Histological regression was reported in 14/16 MTMs (87.4%, *p* = 0.003), including early/inflammatory regression in 2 cases (14.2%), intermediate regression in 7 cases (50%) and late regression in 4 cases (28.5%). Extension of regression was estimated as <75% according to the College of American Pathologists in nine cases (64.2%) and ≥75% in four cases (28.5%).

According to the current staging system (AJCC 8th Edition), the pathological stage at diagnosis was pTx in two regressed melanomas (12.5%), pT1a in eight cases (50%), pT1b in five cases (31.3%) and unknown in one case (6.3%). Overall, four pT1b MTM patients (25%) underwent SLNB, which showed negative results in all cases. In particular, three of them were male with a maximum age of 52 years. SLNs were identified in the axillary region and in the inguinal region in two cases each. One pT1b patient did not undergo SLNB due to the detection of metastasis during staging procedures.

Table 1 shows the histopathological characteristics of the non-MTM control group. Interestingly, the MTMs showed a relatively greater thickness at diagnosis, higher mitotic rate (50% vs. 3%) and regression features (87.4% vs. 53%) in comparison with the control group. All these data when compared with the case group were statistically significant (*p* ≤ 0.05).

In the MTM group, the median follow-up was 58.1 months (range 1.6–174 months). Meanwhile, the median time from primary melanoma diagnosis to first metastasis was 30.1 months (range 2.6–93.2 months). None of our cases presented local relapse, in-transit metastasis or satellitosis. Overall, there were five regional nodal metastases (38.4%) and eight distant metastases (61.5%). Sites of distant metastasis included the lungs (43.7%), brain (43.7%), liver (25%), adrenal gland (12.5%), non-regional lymph nodes (12.5%), skin (6.2%) and bones (6.2%).

Treatment of metastatic disease consisted of combined approaches in most of the cases (56.2%), including surgery, radiotherapy and systemic therapy. Surgery alone, radiotherapy alone and systemic treatment alone (immune checkpoint inhibitors, targeted therapy or chemotherapy) were performed in 25%, 6.2% and 12.5% of the cases, respectively. At the end of the study, five patients (31.2%) were surviving while ten patients (62.5%) had died of melanoma. Only one patient died of another cause.

Younger patients had a poorer prognosis, with a median age at melanoma-related death of 47 years, which was lower than the median age at diagnosis of the primary tumor (49 years). 

## 4. Discussion

In our series, thin melanomas were the most commonly diagnosed lesions (75%), and 1.1% of cases developed regional or distant metastasis, representing 15.4% of the total metastatic melanomas, regardless of thickness. These results are consistent with previous studies [10,11,12]. Dermoscopy remains the preferred pre-operative diagnostic technique for suspicious cutaneous lesions. In the past decades, numerous studies have tried to identify a potential correlation between the dermoscopic and histopathological characteristics of thin melanomas [13,14,15,16,17,18,19,20,21,22]. To our knowledge, no studies have yet investigated the MTM-specific clinical–dermoscopic characteristics. In our retrospective case–control study, we sought to evaluate whether there were clinical or dermoscopic features that could raise a suspicion of potential disease progression by comparing clinical–dermoscopic and histopathological features of MTMs with a control group consisting of non-MTMs with similar demographic characteristics.

Clinically, the most common primary tumor site was the torso (43.7%), as previously described [23,24,25]. Conversely, no gender- or age-specific correlation was found. Interestingly, most of the lesions (73.3%, *p* = 0.002) showed a relatively large diameter (>10 mm), which represents an unusual clinical feature for early-stage melanomas. Notably, all lesions were pigmented, showed a variable grade of dyschromia and presented at least three colors in the same lesion (black, brown, gray, blue, red or white) in 80% (*p* ≤ 0.001) of the cases. Conversely, there were no truly achromic lesions. On the contrary, in the control group, only 29% and 3% of cases had a size of greater than 10 mm and the presence of at least three colors, respectively.

Some authors have analyzed the most common thickness-related dermoscopic characteristics and reported the presence of an atypical pigment network and irregular globules as common features of thinner lesions (≤1 mm) [13,14,15]. The presence of an atypical vascular pattern and regression structures and the absence of a pigment network might be suggestive of thicker lesions (>1 mm) [13,14,15,16,17,18,19,20,21,22]. Notably, in our study, the pigment network was absent in 60% (*p* = 0.001) of the lesions (versus 15% of the control group), while dermoscopic regression structures (white patches and/or blue-gray areas) and an atypical vascular pattern were present in 100% (*p* ≤ 0.001) and 66.6% (*p* ≤ 0.001) of the cases, respectively, representing indicators that should raise a suspicion of this rare subgroup of melanomas. These statistically significant data are truly relevant: in our series, all the thin melanomas that progressed showed dermoscopic parameters of regression and more than half showed an atypical vascular pattern and an absence of the pigment network (dermoscopic parameters normally present in lesions with a thickness > 1 mm).

The dermoscopic features analyzed in this study were homogeneously distributed in all lesions, regardless of the thickness. Several studies reported different Breslow cutoffs by which thin melanomas presented a higher metastatic potential. Some authors reported an association between >0.75 mm thickness and sentinel lymph node positivity [23,26,27], while others considered 0.6 mm, 0.76 mm and ≥0.8 mm as high-risk Breslow cutoffs [10,11,28].

Interestingly, no high-risk melanoma subtypes such as nodular melanomas were included in our series. As expected, relatively higher thicknesses showed a worse prognosis since, among MTMs, 64.2% showed a Breslow thickness ≥ 0.5 mm. In addition, all MTMs were associated with other conventional histopathological features indicative of adverse outcomes, including regression, VGP, ulceration or dermal mitoses [29]. Our results may support the recent proposal of reclassifying thin melanomas (<0.8 mm) in the radial growth phase and characterized by a lack of adverse features (e.g., regression, ulceration and dermal mitoses) as “melanocytic proliferations of low malignant potential” [9]. On the contrary, most TMTs were in the VGP (62% vs. 27% in the control group) and showed regression (87.4% vs. 53% in the control group).

Notably, all cases of patients who underwent SLNB developed distant metastasis without nodal involvement during the follow-up. In contrast to thicker melanomas, the prognostic role of SLNB in thin melanomas is still debated, presenting a positivity rate of 5% (3.2–9.5%) [28,30,31]. Therefore, a risk–benefit assessment for this staging procedure in this subset of patients is mandatory. Given our experience and the low negative predictive value of SLNB in thin melanomas, close monitoring rather than routine SLNB in all pT1a melanomas might be considered.

The brain and lungs were the most common sites of distant metastasis and showed a poor prognosis, which was comparable in terms of frequency and biological aggressiveness to thicker melanomas [32]. All of the patients with distant metastasis received systemic therapy in both metastatic and adjuvant settings. In our study, all patients received chemotherapy before the period of novel therapies, and two patients treated with novel therapies survived, highlighting the revolutionary role of such therapies in advanced melanoma treatment.

The two major limitations of this study are the small sample size and the retrospective data collection. Further prospective multicenter studies are needed, which might consider other important prognostic factors beyond conventional histopathology related to the complex spatial pathobiology of the tumor microenvironment or intrinsic tumoral molecular–genetic features. 

## 5. Conclusions

In conclusion, specific clinical–dermoscopic characteristics such as diameter > 10 mm, at least three colors in the same lesion, the absence of a pigment network, the presence of dermoscopic regression structures and an atypical vascular pattern might be suggestive of a higher metastatic potential in thin melanomas, particularly when coupled with conventional adverse histopathological features (regression, VGP, dermal mitoses). Close and prolonged monitoring of these subsets of patients is recommended. 

## Figures and Tables

**Table 1:** Demographic, clinical and pathological features identified in cases versus controls.

Patients	Cases	Controls	*p*-Value
	N = 16	N = 100	
**Age at diagnosis (yr)**			
Median (IQR)	49 (28–83)	64 (27–91)	
≤50	9 (56.3%)	19 (19%)	**0.003**
>50	7 (43.7%)	81 (81%)	**0.003**
**Gender**			
Female	7 (43.7%)	45 (45%)	0.926
Male	9 (56.3%)	55 (55%)	0.926
**Anatomical site**			
Torso	7 (43.7%)	45 (45%)	0.926
Upper limbs	6 (37.5%)	22 (22%)	0.211
Lower limbs	2 (12.5%)	25 (25%)	0.354
Head/neck	1 (6.3%)	8 (8%)	1.000
**Histological subtype**			
SSM ^a^/Low-CSD melanoma	14 (87.5%)	93 (93%)	0.609
LM ^b^/High-CSD in situ melanoma	2 (12.5%)	7 (7%)	0.609
**Breslow thickness (mm) ^c^**			
Median (IQR)	0.6 (0.2–0.8)	0.15 (0–0.7)	
<0.5	5 (31.2%)	81 (81%)	**<0.001**
≥0.5	9 (56.2%)	9 (9%)	**<0.001**
**Ulceration**			
Present	2 (12.5%)	1 (1%)	**0.044**
Absent	13 (81.2%)	99 (99%)	**0.044**
Missing	1 (6.3%)	0	
**Mitotic rate/mm^2^**			
0	5 (31.5%)	97 (97%)	**<0.001**
1	4 (25%)	3 (3%)	**0.003**
2–4	4 (25%)	0	**<0.001**
Missing	3 (18.5%)	0	
**Growth phase**			
Vertical growth phase (VGP)	10 (62.5%)	27 (27%)	**0.002**
Radial growth phase (RGP)	4 (25%)	73 (73%)	**0.002**
Missing	2 (12.5%)	0	
**Regression**			
Present	14 (87.4)	53 (53%)	**0.003**
<75% ≥75% Missing	9 (64.3%) 4 (28.5%) 1 (7.2%)	34 (64.2%) 19 (35.8%) 0	
Absent	1 (6.3%)	47 (47%)	**0.003**
Missing	1 (6.3%)	0	
**Pathological Stage (AJCC 8th Ed.) ^c^**			
pTx	2 (12.5%)	0	**0.016**
pTis	0	58 (58%)	**<0.001**
pT1a	8 (50%)	41 (41%)	0.368
pT1b	5 (31.2%)	1 (1%)	**<0.001**
Missing	1 (6.3%)	0	
**SLNB ^d^**			
Not performed	12 (75%)	99 (99%)	
Negative	4 (25%)	1 (1%)	

^a^ SSM: superficial spreading melanoma; CSD: cumulative sun damage; ^b^ LM: lentigo maligna; ^c^ AJCC: American Joint Committee on Cancer; ^d^ SLNB: sentinel lymph node biopsy. A two-tailed *p*-value of less than 0.05 constituted statistical significance.

**Table 2:** Clinical and dermoscopic features identified in metastasizing thin melanomas (MTMs) versus controls (non-MTM).

Patients	Cases N = 16	Controls N = 100	*p*-Value
**Clinical–dermoscopic features**			
≥3 colors	12 (80%)	3 (3%)	**<0.001**
Diameter > 10 mm	11 (73.3%)	29 (29%)	**0.002**
White patch	11 (73.3%)	43 (43%)	0.055
Atypical vascular patterns	10 (66.5%)	15 (15%)	**<0.001**
Blue-gray areas	9 (60%)	27 (27%)	**0.038**
Absence of pigment network	9 (60%)	15 (15%)	**0.001**
Blue-white veil	3 (20%)	5 (5%)	0.079
Regression structures (white patch or blue-gray areas)	16 (100%)	45 (45%)	**<0.001**

A two-tailed *p*-value of less than 0.05 constituted statistical significance.

## Data Availability

Data available on request due to privacy/ethical restrictions.

## References

[1] Elder D.E. (2011). Thin melanoma. Arch. Pathol. Lab. Med..

[2] Nazzaro G., Passoni E., Pozzessere F., Maronese C.A., Marzano A.V. (2022). Dermoscopy Use Leads to Earlier Cutaneous Melanoma Diagnosis in Terms of Invasiveness and Size? A Single-Center, Retrospective Experience. J. Clin. Med..

[3] Karakousis G., Gimotty P.A., Bartlett E.K., Sim M.S., Neuwirth M.G., Fraker D., Czerniecki B.J., Faries M.B. (2017). Thin Melanoma with Nodal Involvement: Analysis of Demographic, Pathologic, and Treatment Factors with Regard to Prognosis. Ann. Surg. Oncol..

[4] Claeson M., Baade P., Brown S., Soyer H.P., Smithers B.M., Green A.C., Whiteman D.C., Khosrotehrani K. (2020). Clinicopathological factors associated with death from thin (≤1·00 mm) melanoma. Br. J. Dermatol..

[5] Gershenwald J.E., Scolyer R.A., Hess K.R., Sondak V.K., Long G.V., Ross M.I., Lazar A.J., Faries M.B., Kirkwood J.M., McArthur G.A. (2017). Melanoma staging: Evidence-based changes in the American Joint Committee on Cancer eighth edition cancer staging manual. CA Cancer J. Clin..

[6] Mihic-Probst D., Shea C., Duncan L., de la Fouchardiere A., Landman G., Landsberg J., ven den Oord J., Lowe L., Cook M.G., Yun S.J. (2016). Update on Thin Melanoma: Outcome of an International Workshop. Adv. Anat. Pathol..

[7] Egger M.E. (2021). Prognosis in Thin Melanoma Patients: Is Slightly Less than Excellent Still Okay?. Ann. Surg. Oncol..

[8] Kaufmann C., Kempf W., Mangana J., Cheng P., Emberger M., Lang R., Kaiser A.K., Lattmann E., Levesque M., Dummer R. (2020). The role of cyclin D1 and Ki-67 in the development and prognostication of thin melanoma. Histopathology.

[9] Elder D.E., Massi D., Scolyer R.A. (2018). WHO Classification of Skin Tumours.

[10] Isaksson K., Mikiver R., Eriksson H., Lapins J., Nielsen K., Ingvar C., Lyth J. (2021). Survival in 31 670 patients with thin melanomas: A Swedish population-based study. Br. J. Dermatol..

[11] Richetta A.G., Valentini V., Marraffa F., Paolino G., Rizzolo P., Silvestri V., Zelli V., Carbone A., Di Mattia C., Calvieri S. (2018). Metastases risk in thin cutaneous melanoma: Prognostic value of clinical-pathologic characteristics and mutation profile. Oncotarget.

[12] Kalady M.F., White R.R., Johnson J.L., Tyler D.S., Seigler H.F. (2003). Thin melanomas: Predictive lethal characteristics from a 30-year clinical experience. Ann. Surg..

[13] Podolec K., Bronikowska A., Pirowska M., Wojas-Pelc A. (2020). Dermoscopic features in different dermatopathological stages of cutaneous melanomas. Postepy Dermatol. Alergol..

[14] Silva V.P., Ikino J.K., Sens M.M., Nunes D.H., Di Giunta G. (2013). Dermoscopic features of thin melanomas: A comparative study of melanoma in situ and invasive melanomas smaller than or equal to 1mm. An. Bras. Dermatol..

[15] Ungureanu L., Şenilă S., Dănescu S., Rogojan L., Cosgarea R. (2013). Correlation of dermatoscopy with the histopathological changes in the diagnosis of thin melanoma. Rom. J. Morphol. Embryol..

[16] Guitera P., Li L.X., Crotty K., Fitzgerald P., Mellenbergh R., Pellacani G., Menzies S.W. (2008). Melanoma histological Breslow thickness predicted by 75-MHz ultrasonography. Br. J. Dermatol..

[17] Argenziano G., Fabbrocini G., Carli P., De Giorgi V., Delfino M. (1997). Epiluminescence microscopy: Criteria of cutaneous melanoma progression. J. Am. Acad. Dermatol..

[18] Lorentzen H.F., Weismann K., Larsen F.G. (2001). Dermatoscopic prediction of melanoma thickness using latent trait analysis and likelihood ratios. Acta Derm. Venereol..

[19] Hayashi K., Koga H., Uhara H., Saida T. (2009). High-frequency 30-MHz sonography in preoperative assessment of tumor thickness of primary melanoma: Usefulness in determination of surgical margin and indication for sentinel lymph node biopsy. Int. J. Clin. Oncol..

[20] Argenziano G., Fabbrocini G., Carli P., De Giorgi V., Delfino M. (1999). Clinical and dermatoscopic criteria for the preoperative evaluation of cutaneous melanoma thickness. J. Am. Acad. Dermatol..

[21] Carli P., de Giorgi V., Palli D., Giannotti V., Giannotti B. (2000). Preoperative assessment of melanoma thickness by ABCD score of dermatoscopy. J. Am. Acad. Dermatol..

[22] Stante M., De Giorgi V., Cappugi P., Giannotti B., Carli P. (2001). Non-invasive analysis of melanoma thickness by means of dermoscopy: A retrospective study. Melanoma Res..

[23] Steding-Jessen M., Hölmich L.R., Chakera A.H., Klausen S., Hovaldt H.B., Møller H. (2022). Thin or early melanoma, risk factors and associated mortality. Dan. Med. J..

[24] Guitart J., Lowe L., Piepkorn M., Prieto V.G., Rabkin M.S., Ronan S.G., Shea C.R., Tron V.A., White W., Barnhill R.L. (2002). Histological characteristics of metastasizing thin melanomas: A case-control study of 43 cases. Arch. Dermatol..

[25] Vilmer C., Bailly C., Le Doussal V., Lasry S., Guerin P., Delaunay M.M., Mandard A.M. (1996). Thin melanomas with unusual aggressive behavior: A report on nine cases. Melanoma Group of French Federation of Cancer Centers. J. Am. Acad. Dermatol..

[26] Maurichi A., Miceli R., Camerini T., Mariani L., Patuzzo R., Ruggeri R., Gallino G., Tolomio E., Tragni G., Valeri B. (2014). Prediction of survival in patients with thin melanoma: Results from a multi-institution study. J. Clin. Oncol..

[27] Cordeiro E., Gervais M.K., Shah P.S., Look Hong N.J., Wright F.C. (2016). Sentinel Lymph Node Biopsy in Thin Cutaneous Melanoma: A Systematic Review and Meta-Analysis. Ann. Surg. Oncol..

[28] Murali R., Haydu L.E., Long G.V., Quinn M.J., Saw R.P., Shannon K., Spillane A.J., Stretch J.R., Kefford R.F., Thompson J.F. (2012). Clinical and pathologic factors associated with distant metastasis and survival in patients with thin primary cutaneous melanoma. Ann. Surg. Oncol..

[29] Swetter S.M., Thompson J.A., Albertini M.R., Barker C.A., Baumgartner J., Boland G., Chmielowski B., Di Maio D., Durham A., Fields R.C. (2021). NCCN Guidelines^®^ Insights: Melanoma: Cutaneous, Version 2.2021. J. Natl. Compr. Cancer Netw..

[30] Hieken T.J., Grotz T.E., Comfere N.I., Inselman J.W., Habermann E.B. (2015). The effect of the AJCC 7th edition change in T1 melanoma substaging on national utilization and outcomes of sentinel lymph node biopsy for thin melanoma. Melanoma Res..

[31] Oude Ophuis C.M., Louwman M.W., Grünhagen D.J., Verhoef K., van Akkooi A.C. (2017). Implementation of the 7th edition AJCC staging system: Effects on staging and survival for pT1 melanoma. A Dutch population based study. Int. J. Cancer.

[32] Gorka E., Fabó D., Gézsi A., Czirbesz K., Liszkay G. (2016). Distance from Primary Tumor Is the Strongest Predictor for Early Onset of Brain Metastases in Melanoma. Anticancer Res..

